# Antifungal and carboxylesterase-producing bacteria applied into corn silage still affected the fermented total mixed ration

**DOI:** 10.5713/ab.22.0232

**Published:** 2022-11-14

**Authors:** Dimas Hand Vidya Paradhipta, Myeong Ji Seo, Seung Min Jeong, Young Ho Joo, Seong Shin Lee, Pil Nam Seong, Hyuk Jun Lee, Sam Churl Kim

**Affiliations:** 1 Department of Animal Nutrition and Feed Science, Faculty of Animal Science, Universitas Gadjah Mada, Yogyakarta 55281, Indonesia; 2 Division of Applied Life Science (BK21Four, Insti. of Agri. & Life Sci.), Gyeongsang National University, Jinju 52828, Korea; 3 Animal Nutrition and Physiology Division, National Institute of Animal Science, RDA, Wanju 55365, Korea

**Keywords:** Antifungal Substance, Carboxylesterase, Corn Silage, Fermented Total Mixed Ration, Microbial Inoculant

## Abstract

**Objective:**

This study investigated the effects of corn silage as a source of microbial inoculant containing antifungal and carboxylesterase-producing bacteria on fermentation, aerobic stability, and nutrient digestibility of fermented total mixed ration (FTMR) with different energy levels.

**Methods:**

Corn silage was used as a bacterial source by ensiling for 72 d with an inoculant mixture of *Lactobacillus brevis* 5M2 and *L. buchneri* 6M1 at a 1:1 ratio. The corn silage without or with inoculant (CON vs MIX) was mixed with the other ingredients to formulate for low and high energy diets (LOW vs HIGH) for Hanwoo steers. All diets were ensiled into 20 L mini silo (5 kg) for 40 d in quadruplicate.

**Results:**

The MIX diets had lower (p<0.05) acid detergent fiber with higher (p<0.05) *in vitro* digestibilities of dry matter and neutral detergent fiber compared to the CON diets. In terms of fermentation characteristics, the MIX diets had higher (p<0.05) acetate than the CON diets. The MIX diets had extended (p<0.05) lactic acid bacteria growth at 4 to 7 d of aerobic exposure and showed lower (p<0.05) yeast growth at 7 d of aerobic exposure than the CON diets. In terms of rumen fermentation, the MIX diets had higher (p<0.05) total fermentable fraction and total volatile fatty acid, with lower (p<0.05) pH than those of CON diets. The interaction (p = 0.036) between inoculant and diet level was only found in the immediately fermentable fraction, which inoculant was only effective on LOW diets.

**Conclusion:**

Application of corn silage with inoculant on FTMR presented an antifungal effect by inhibiting yeast at aerobic exposure and a carboxylesterase effect by improving nutrient digestibility. It also indicated that fermented feedstuffs could be used as microbial source for FTMR. Generally, the interaction between inoculant and diet level had less effect on this FTMR study.

## INTRODUCTION

Fermented total mixed rations (FTMR) are formulated diets according to the nutrient requirement of ruminants made by fermenting the combination of protein, energy, roughage, mineral, and vitamin sources [1]. Fermentation of the completed ration shows several beneficial effects, such as avoiding nutrient loss and improving the palatability of by-products [1–4]. In practical field applications, several problems arise with the FTMR, including low digestibility and fungal contamination, are still found. These problems can occur because farmers commonly use poor-quality roughages to reduce the feed cost, such as straw or other post-harvest agriculture by-products such as fiber sources [1,5,6]. These poor-quality roughages have a high concentration of lignocellulose, which can decrease the nutrient digestibility of FTMR [7,8]. Besides that, roughage or other feedstuffs for making rations can be potentially contaminated with undesirable microbes due to the environmental conditions of the farm or warehouse [9], which can decrease the fermentation quality of FTMR.

Several strains of lactic acid bacteria (LAB) were reported to release specific metabolite products, such as antifungal substances [10] or fibrolytic enzymes [11,12]. In silage application, antifungal-producing LAB have been shown to decrease the contamination of undesirable microbes [10, 13,14], while carboxylesterase-producing LAB could increase the nutrient digestibility [11,12,14]. Mainly in roughages, lignocellulose is the limiting factor of digestibility due to the limitation of lignocellulose degrading enzymes in the rumen [8]. Cellulose and hemicellulose are bounded by a lignin linkage to configure into lignocellulose. Carboxylesterase can breakdown the lignin linkage, which consists of an ester compound, and then release hemicellulose and cellulose [7,8]. Similar to silage, LAB also have been applied to improve the fermentation quality of FTMR [3–6]. However, there are limited studies on the application of LAB with a specific purpose, such as producing antifungal or carboxylesterase activities in FTMR. Moreover, none of current studies have investigated the effects of ration ingredients as a source of microbial inoculant for FTMR. In practical field applications, silage is commonly used as an ingredient of FTMR as a fiber source, in which dominant microbes such as LAB may still be alive in the silage and can control the fermentation of FTMR.

New dual-purpose LAB consisting of *Lactobacillus brevis* 5M2 and *L. buchneri* 6M1 were isolated from corn silage in our previous study [11]. Both *L. brevis* 5M2 and *L. buchneri* 6M1 improved the antifungal activities against *Fusarium graminearum* and the carboxylesterase activities for aerobic stability and fiber digestibility, respectively. Furthermore, the new dual-purpose LAB were used as an inoculant in corn silage and resulted in the improvement of silage quality by presenting antifungal and carboxylesterase effects [11]. Additionally, microbial inoculants have become an important necessity for the FTMR industry. The use of silage as an ingredient in FTMR potentially can replace the use of microbial inoculants and help to reduce the production cost. This silage still contains the applied microbes after the silo is opened. The presence of new dual-purpose LAB from corn silage is expected to be a microbial source that produces antifungal and carboxylesterase activities in FMTR when used as an ingredient. Therefore, the aim of this study was to investigate the effects of corn silage as a source of microbial inoculant on the fermentation quality, aerobic stability, and nutrient digestibility of FTMR with two different energy levels.

## MATERIALS AND METHODS

### Fermented total mixed ration

In a previous study [11], baled corn silages (500 kg) were ensiled for 72 d with or without microbial inoculant application. The silage inoculant used was a mixture of *L. brevis* 5M2 KACC 92268P and *L. buchneri* 6M1 KACC 92269P (Korean Culture Center of Microorganism, Seoul, Korea) at a 1:1 ratio. A total of 500 mL of inoculant was sprayed into the corn forage with an application rate of 1×10^5^ colony forming units (cfu)/g fresh forage. Both silages without or with inoculant had a similar pH (3.80 vs 3.78), lactate (130 vs 129 g/kg DM), and LAB (6.82 vs 6.89 log10 cfu/g), but slight differences on acetate (20.9 vs 24.9 g/kg DM) and yeast (5.89 vs 5.79 log10 cfu/g). In FTMR production, the corn silages without (CON) or with inoculant (INO) were mixed with the other ingredients to adjust two different energy levels consisting of the low energy diet for growing phase (LOW) and the high energy diet for finishing phase (HIGH) for Hanwoo steers. All diets were formulated iso-nitrogenous and iso-caloric to meet the nutrient requirements according to the Korean Feeding Standard for Korean Cattle [15]. The ingredients of diets before fermentation are presented in Table 1. The mixture from each diet was ensiled into 20 L mini silos (5 kg) for 40 d in quadruplicate. The fresh diets just before fermentation and the FTMR after fermentation were sub-sampled 2 kg for chemical compositions and *in vitro* rumen digestibility analyses. Also, 20 g FTMR was sub-sampled and blended with 200 mL of sterile ultrapure water for 30 s, and then filtered through two layers of cheesecloth to make an extraction [16,17]. The fresh FTMR extraction (2 mL), with no cold storage, was sub-sampled to determine the microbial count. After measuring pH, the FTMR extraction was stored at −70°C until the analyses of fermentation characteristics such as pH, ammonia-N, lactate, and volatile fatty acid (VFA) [16,17]. The aerobic deterioration was measured using 1 kg of sub-sampled FTMR in aerobic conditions.

### Chemical compositions

One kilogram of sub-sampled diets before (TMR) and after fermentation (FTMR) were dried at 65°C for 48 h and ground using a cutting mill (Shinmyung Electric Co., Ltd, Gimpo, Korea) to pass a 1-mm screen for the measurements of chemical compositions and *in vitro* digestibility. The dry matter (DM) concentrations of the diets were measured by drying 10 g samples at 105°C for 24 h in an air-forced dry oven (OF-22GW; Jeio Tech, Seoul, Korea). Samples from DM measurement were placed into a muffle furnace at 550°C for 5 h to determine crude ash (CA). The procedure of Kjeldahl (method 984.13 of AOAC [18]) was used to determine crude protein (CP) using a N analyzer (B-324, 412, 435, and 719 S Titrino; BUCHI, Flawil, Switzerland). The procedure of Soxhlet (method 920.39 of AOAC [18]) was used to determine ether extract (EE). The neutral detergent fiber (NDF) and acid detergent fiber (ADF) were determined by using an Ankom 200 fiber analyzer (Ankom Technology, Macedon, NY, USA) according to the protocol of AOAC (method 2002.04 and method 973.18, respectively) [18]. In addition, heat stable amylase was applied in NDF analysis. The hemicellulose (HEMI) was determined by calculating the differences between NDF and ADF. The ground samples at 0.5 g were put into filter bags (F57 ANKOM bag; Ankom Technology, USA) for the preparation of ruminal *in vitro* incubation. *In vitro* digestibilities of DM (IVDMD) and NDF (IVNDFD) were determined after 48 h of ruminal incubation by the method of Tilley and Terry [19] using an Ankom Daisy (Ankom Technology, USA). Then, the IVDMD and the IVNDFD were calculated as a percentage of DM (%, DM).

### Fermentation characteristics

The pH of FTMR was determined using a pH meter (SevenEasy; Mettler Toledo, Greifensee, Switzerland). The colorimetric method described by Chaney and Marbach [20] was used to determine the concentration of ammonia-N. The FTMR extraction was centrifuged at 5,645×*g* for 15 min to separate the supernatant and residue. A collected supernatant was used for lactate and VFA analyses. The concentrations of lactate and VFA were determined using a HPLC (L-2200; Hitachi, Tokyo, Japan) fitted with a UV detector (L-2400; Hitachi, Japan) and a column (Metacarb 87H; Varian, Palo Alto, CA, USA) according to the method described by Muck and Dickerson [21].

### Microbial counts

The fresh FTMR extract (first dilution) was made into several dilutions (10^−4^ to 10^−8^) to determine the microbial counts for LAB, bacilli, yeast, and mold. The FTMR extract was planted into selective agar medium in triplicate. The lactobacilli Man Rogosa Sharpe agar media (MRS; Difco, Detroit, MI, USA) was used for LAB count, Luria-Bertani agar media (LB; Difco, USA) was used for *Bacillus* count, and potato dextrose agar media (PDA; Difco, USA) was used for counting fungi, consisted of yeast and mold. The MRS agar plates were placed in a CO_2_ incubator (Thermo Scientific, Waltham, MA, USA) at 30°C for 48 h, while PDA and LB agar plates were incubated at 30°C for 72 h in an aerobic incubator (Johnsam Corp., Boocheon, Korea) [11,12]. Visible colonies were counted from the plates. The number of cfu was expressed per gram of FTMR (log 10 cfu/g).

### Aerobic stability

The estimation of aerobic stability was conducted in field conditions during the cold season, which had an ambient temperature ranging 9.2°C to 10.6°C. The sub-sampled FTMR was located in a polystyrene box during aerobic conditions. The temperature was recorded by a thermocouple wire sensor (MORGAN TR-60CH, Hong Kong, China) that was placed at the geometric center of each sample. Two sensors as laboratory replicates were used for each silo with three sensors to record the room temperature. Data were collected every 30 min with a computer. Aerobic stability was calculated by the time (h) before a 2°C increase in FTMR temperature above the ambient temperature [11,14]. On 1, 2, 3, 4, and 7 d of aerobic exposure, 50 g of FTMR was sub-sampled to measure changes in pH and microbes [11].

### *In vitro* rumen incubation

The procedure of animal care for maintaining cannulated Hanwoo heifers was approved by the Animal Ethical Committee of Gyeongsang National University, Jinju, Korea (GNU-191011-E0050). Before morning feeding, the rumen fluid was collected from two non-pregnant cannulated Hanwoo heifers. The Hanwoo heifers were fed rice straw and commercial concentrate mixed at an 8:2 ratio. The collected rumen fluid was filtered via two layers of cheesecloth. Rumen buffer was prepared by mixing rumen fluid with anaerobic culture medium at a 1:2 ratio as described by Adesogan et al [22]. The ground sample was weighted at 0.5 g and then put into an incubation bottle. Rumen buffer at 40 mL was added into each incubation bottle. To reach anaerobic conditions, all incubation bottles were gassed with CO_2_ and closed tightly. Four replications for each treatment were prepared along with two blanks. All incubation bottles were placed at 39°C in an incubator (OF-22GW; Jeio Tech, Korea). The gas pressure was monitored with a computer every 30 min for 48 h using a wireless automated system (ANKOM^RF^; ANKOM Technology, USA) to calculate the fermentation kinetics in the rumen [22]. The kinetics were generated using PROC NLIN of Statistical Analysis Software (SAS, Version 9. Cary, NC, USA) [23] to fit with the model of McDonald [24] following:


$$\text{Y}=\text{A}+\text{B }(1-{\text{e}}^{-\text{c}\;(\text{t}-\text{L})})\;\text{for t}>\text{L}$$

where A is the immediately fermentable fraction; B is the potentially fermentable fraction; A+B is total fermentable fraction; C is the fractional fermentation rate; L is the lag phase; and t is time of incubation (h).

After 48 h, all incubation bottles were opened and transferred to 50 mL conical tubes to separate the residue and supernatant (rumen buffer) through centrifugation at 2,568×*g* for 15 min (Supra 21k; Hanil Electric Corporation, Seoul, Korea; with rotor A50S-6C No.6). The supernatant was used to analyze rumen fermentation indices such as rumen pH, ammonia-N, and VFA. The protocol for rumen pH, ammonia-N, and VFA analyses was same as described in the previous section.

### Statistical analysis

The experiment was conducted by a 2 (Inoculant; CON vs MIX)×2 (Diet level; LOW vs HIGH) factorial design with four replicates per treatment and all data were analyzed PROC MIXED to test the effects of inoculant, diet level, and its interaction (inoculant×diet level). The model was *Y_ijk_* = *μ*+*α_i_*+*β_j_*+(*αβ*)*_ij_*+*e_ijk_*, where *Y_ijk_* = response variable, *μ* = overall mean, *α_i_* = the effect of inoculant, *β_j_* = the effect of diet level, (*αβ*)*_ij_* = the interaction effect of inoculant and diet level, *e_ijk_* = error term. For pH and microbial counts during aerobic exposure, data were analyzed as a factorial design with a 2 (Inoculant; CON vs MIX)×2 (Diet level; LOW vs HIGH)×6 (Aerobic day; 0 vs 1 vs 2 vs 3 vs 4 vs 7 d) arrangement using PROC MIX. Its general model was *Y_ijkl_* = *μ*+*α_i_*+*β_j_*+*Ɣ_k_*+(*αβ*)*_ij_*+(*αƔ*)*_ik_*+(*βƔ*)*ik*+(*αβƔ*)*_ijk_*+*e_ijkl_*, where *Y_ijkl_* = response variable, *μ* = overall mean, *α_i_* = the effect of inoculant, *β_j_* = the effect of diet level, *Ɣ_k_* = the effect of aerobic hour, (*αβ*)*_ij_* = the interaction effect of inoculant and diet level, (*αƔ*)*_ik_* = the interaction effect of incoulant and aerobic hour, (*βƔ*)*_jk_* = the interaction effect of diet level and aerobic hour, (*αβƔ*)*_ijk_* = the interaction effect of inoculant, diet level, and aerobic hour, *e_ijkl_* = error term. The polynomial contrasts analysis by PROC general linear model was used to evaluate the effects of increasing aerobic day (linear or quadratic) on pH and microbial counts during aerobic exposure. In addition, orthogonal coefficients for linear, quadratic, and cubic contrast were adjusted to account for the unequal spacing of aerobic days (0, 1, 2, 3, 4, and 7 d) with PROC IML before testing the polynomial contrast. Mean comparisons were performed by Tukey’s test and the significant differences were declared at p≤0.05. All statistical analyses in the present study were performed according to the protocol of SAS program [23].

## RESULTS

### Chemical compositions

The chemical compositions of the CON and MIX diets before fermentation were not different (Table 2). On the other hand, the HIGH diets had higher concentrations of (p≤0.001) DM, CP, IVDMD, IVNDFD, and total digestible nutrients with lower (p<0.010) concentrations of EE, CA, NDF, ADF, and HEMI than those of LOW diets.

After fermentation, MIX had a lower ADF concentration (p = 0.032; 21.7% vs 23.7% and 15.2% vs 16.0%) with higher IVDMD (p<0.001; 62.0% vs 59.9% and 69.0% vs 65.9%) and IVNDFD (p = 0.001; 52.2% vs 49.7% and 58.2% vs 55.2%) than those of CON in both the HIGH and LOW diets, but other parameters were not affected by the inoculant (Table 3). The HIGH diets had higher (p<0.001) concentrations of DM, CP, IVDMD, and IVNDFD with lower (p<0.001) concentrations of EE, CA, NDF, ADF, and HEMI compared to the LOW diets.

### Fermentation characteristics

The MIX had a higher acetate concentration (p = 0.010; 2.38% vs 2.13% and 4.01% vs 3.79%) than CON in both the LOW and HIGH diets, while the pH, ammonia-N, other VFA profiles, lactate to acetate ratio, and microbial counts were not affected by the inoculant (Table 4). The HIGH diets had higher (p<0.05) pH and concentrations of ammonia-N, lactate, and acetate, but lower lactate to acetate ratio and counts of LAB and yeast than those of the LOW diets. The *Bacillus* count was not affected by the inoculant and diet level, while mold was not detected after silo was opened and during aerobic exposure.

### Aerobic stability and microbial changes

**Figure 1:**
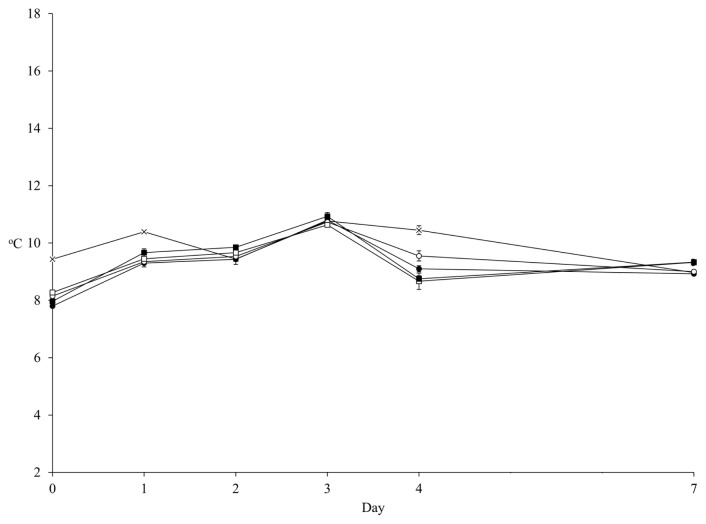
Temperature of FTMR during aerobic exposure. The ambient temperature (×); LOW diet with corn silage applied no inoculant (○) or corn silage applied mixture of *L. brevis* 5M2 and *L. buchneri* 6M1 (●); and HIGH diet with corn silage applied no inoculant (□) or corn silage applied inoculant mixture of *L. brevis* 5M2 and *L. buchneri* 6M1 (■). Error bar indicated standard error. FTMR, fermented total mixed ration.

**Figure 2:**
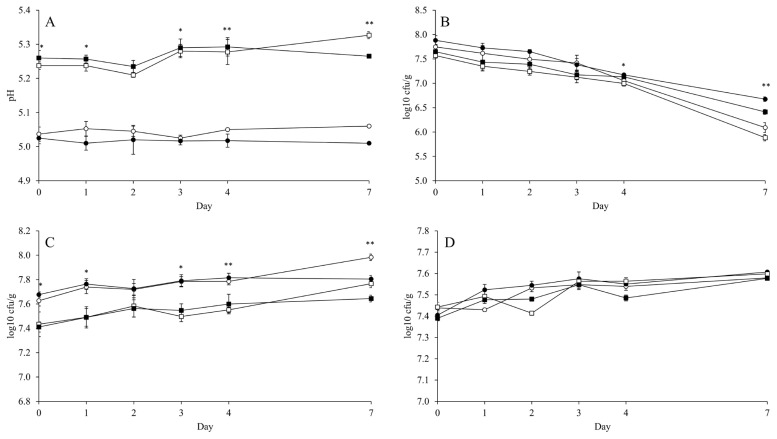
Changes of pH (A), lactic acid bacteria (B), *Bacillus* (C), and yeast (D) of fermented total mixed ration during aerobic exposure. LOW diet with corn silage applied no inoculant (○) or corn silage applied mixture of *L. brevis* 5M2 and *L. buchneri* 6M1 (●); and HIGH diet with corn silage applied no inoculant (□) or corn silage applied inoculant mixture of *L. brevis* 5M2 and *L. buchneri* 6M1 (■). Values differ between groups within same day * p<0.05 and ** p<0.01. Error bar indicated standard error. The contrast of pH, lactic acid bacteria, *Bacillus*, and yeast were presented at appendix.

The mean of ambient temperature during 7 d of aerobic exposure was 9.91°C and the temperatures of all diets were not 2°C higher than the ambient temperature during observation (Figure 1). Thus, the aerobic stability of FTMR could not be determined in the present study. Nevertheless, the counts of LAB, yeast, and *Bacillus* increased linearly (p<0.001) by aerobic day, while the pH maintained a stable pattern (Figure 2; Supplementary Table S1). The inoculant had no effect on pH over 4 d of aerobic exposure. The MIX presented a lower pH (p<0.05; 5.01 vs 5.06 and 5.27 vs 5.33) than CON in both the HIGH and LOW diets at 7 d of aerobic exposure (Figure 2A). Also, the inoculant had no effect on LAB count over 3 d of aerobic exposure, but MIX presented a higher LAB count than CON in both the HIGH and LOW diets at 4 d (p<0.05; 7.17 vs 7.07 and 7.13 vs 7.00 log10 cfu/g) and 7 d (p<0.05; 6.67 vs 6.09 and 6.45 vs 5.88 log10 cfu/g) of aerobic exposure (Figure 2B). The count of *Bacillus* was not affected by the inoculant and diet level during aerobic exposure (Figure 2C). Yeast count was not affected by the inoculant over 4 d of aerobic exposure, but MIX had a lower (p<0.05; 7.81 vs 7.98 and 7.64 vs 7.77 log10 cfu/g) count than CON in both HIGH and LOW diets at 7 d of aerobic exposure (Figure 2D). Based on the mean of microbial count, MIX had a higher LAB count (p = 0.013; 7.41 vs 7.24 and 7.20 vs 7.03 log10 cfu/g) during aerobic exposure than CON (Table 5). However, the inoculant had no effect on the mean of pH and the means of *Bacillus* and yeast counts during aerobic exposure. Generally, the diet level had an effect (p≤0.001) on all microbes, except on *Bacillus*.

### Rumen fermentation kinetics estimated from *in vitro* gas production

The interaction between the inoculant and diet level was found in the immediately fermentable fraction (Table 6) where MIX only increased in the LOW diet (p = 0.036; 1.95 vs 1.77 mL/g). The MIX in both diets had higher the potentially fermentable fraction (p = 0.018; 2.47 vs 2.36 mL/g and 3.49 vs. 3.12 mL/g) and the total fermentable fraction (p = 0.009; 4.41 vs 4.16 mL/g and 5.42 vs 5.12 mL/g) compared to those of CON in both diets, while and the lag phase were not affected by the inoculant. The HIGH diet was reported a higher (p<0.001) potentially fermentable fraction and total fermentable fraction, and a lower (p<0.001) lag phase than those of LOW diet.

### Rumen fermentation indices

In the rumen fermentation indices, MIX had lower pH (p = 0.034; 6.84 vs 6.88 and 6.68 vs 6.75) and iso-valerate concentration (p = 0.040; 2.88% vs 3.27% and 2.67% vs 2.81%), but a higher total VFA concentration (p = 0.014; 114.4 vs 107.9 m*M*/L and 129.2 vs 119.4 m*M*/L) than those of CON in HIGH and LOW diets (Table 7). The concentration of ammonia-N and other VFA profiles were not affected by the inoculant. The HIGH diet had lower (p<0.05) pH and acetate concentration, but higher (p<0.05) concentrations of ammonia-N, total VFA, and butyrate than those of LOW diet.

## DISCUSSION

In the present study, the application of corn silage with dual-purpose LAB decreased ADF concentration and increased IVDMD and IVNDFD concentrations in both FTMR diets. These results were in agreement with several previous studies that reported similar results by application of carboxylesterase-producing LAB to corn silage or rye silage [11,12,14]. These results indicated that *L. brevis* 5M2 and *L. buchneri* 6M1 from corn silage could be a microbial source to control the fermentation of FTMR in the present study. Both *L. brevis* 5M2 and *L. buchneri* 6M1 produced carboxylesterase during fermentation and could degrade the lignocellulose, which might also decrease ADF concentration after silo opening. The degradation of lignocellulose could release the free structural carbohydrates from the lignin linkage that can increase the accessibility of fibrolytic enzymes by rumen microbes to degrade cellulose and hemicellulose [7,8]. Thus, it caused increases of IVDMD and IVNDFD in the present study.

In terms of the fermentation characteristics, pH was not affected by the inoculant in the present study, possibly due to a similar concentration of total organic acid (lactate and acetate) in CON and in MIX (data was not shown; p = 0.102; 4.63% vs 4.77%). This was also a reason why the inoculant had no effect on ammonia-N concentration. Although it produced higher lactate and acetate concentrations, the HIGH diet presented a higher pH than the LOW diet. This result occurred because the HIGH diet produced a higher ammonia-N concentration, which acted as a buffer to inhibit pH decreases [25,26]. A high concentration of ammonia-N in the HIGH diet could be caused by a high CP concentration before fermentation (Table 2). The ammonia-N concentration was a result of CP degradation during fermentation, where the higher CP concentration before ensiling could result in higher ammonia-N concentration after ensiling [25,26].

Application of corn silage with dual-purpose LAB also increased the acetate concentration of FTMR. This result could be caused by a high acetate concentration in a corn silage applied inoculant, but this was not a major reason as only a small proportion (<15%) of corn silage was applied to the FTMR. Another reason is that the corn silage was treated with *L. brevis* 5M2 and *L. buchneri* 6M1 as heterofermentative LAB, which might influence the high acetate production found in the MIX diets. In addition, lactate was numerically lower in the MIX diets than in the CON diets that might be caused by the conversion of lactate to acetate by the heterofermentative LAB [16,25]. Fermentation characteristics of the present study supported the results of our previous study, where inoculation of a baled corn silage with *L. brevis* 5M2 and *L. buchneri* 6M1 improved the acetate concentration [11]. In general, the HIGH diets produced higher concentrations of lactate and acetate than the LOW diets because it provided more energy sources that could be used by LAB to produce more organic acids [25,27].

Application of corn silage with *L. brevis* 5M2 and *L. buchneri* 6M1 did not improve the LAB count of FTMR at silo opening. This result was similar with our previous study, where inoculation of corn silage with mixed *L. brevis* 5M2 and *L. buchneri* 6M1 also had no effect on LAB count at silo opening [11]. There are several factors that affect the number of LAB during ensiling including WSC, anaerobe conditions, and moisture content [25]. The inoculation of LAB did not result in an increase of its populations by the end of ensiling even though it resulted in the improvement of fermentation characteristics [11]. The count of LAB could be similar, but the species might be different by inoculation. In the present study, the improvement of acetate concentration by the inoculant did not decrease the yeast count of FTMR at silo opening, which was similarly reported in several previous studies [3,5]. A good fermentation process in all diets might inhibit mold growth effectively in the present study. It might also be a reason why there was no effects of inoculant and diet level on *Bacillus* and yeast growths. Furthermore, the good fermentation process in the present study could be indicated by the low ammonia-N (<0.01%) and having no butyrate detected in all diets, which supported the opinion of a previous study [25]. On the other hand, the application of diets with different energy levels seemed to be the main factor that influenced the counts of LAB and yeast in the present study. The HIGH diets presented lower LAB and yeast counts due to lower moisture content and higher organic acid concentration, respectively [25,28,29].

All diets presented aerobic stability more than 7 d in the present study based on the observations of temperature, pH, and microbial count, which were generally in stationary phase. The stationary phase of pH during aerobic exposure indicated fewer microbial changes, which supported a longer shelf life of FTMR during the feed-out phase. Previous studies reported that the aerobic stability of FTMR could be varied from 3 to 7 d depending on the diet composition and moisture content, where high moisture and WSC contents would accelerate the rapid spoilage during aerobic conditions [2,27]. Also, the low temperature of environment could increase the aerobic stability of FTMR due to the inhibition of undesirable microbes at low temperatures [11,25]. In the present study, the determination of aerobic stability during the cold season was conducted following the real application of our FTMR on the farm. Paradhipta et al [17] similarly reported that yeasts grew slowly and molds were not detected on sorughum-sudangrass silage during 8 d of aerobic exposure. In terms of application in the field, the low temperature during aerobic exposure occurred because the FTMR was opened in farm conditions during the cold season. During aerobic exposure, the presence of oxygen decreased linearly the LAB count by the hour [3,25]. However, the MIX diets extended LAB count from 4 to 7 d, which indicated that *L. brevis* 5M2 and *L. buchneri* 6M1 in the MIX diets might have higher aerobic tolerance than the epiphytic bacteria in the CON diets. The mean of microbial count during aerobic exposure also showed that the application of MIX diets extended the counts of LAB in both of diets. This result also supported our previous study, which reported a similar result with corn silage [11]. For the aerobic microbes, the growths of yeast and *Bacillus* in all diets increased linearly by the hour, which supported the results of previous studies [3,25]. The MIX diets presented a lower yeast count than CON diets at 7 d as the result of antifungal activity from *L. brevis* 5M2 and *L. buchneri* 6M1, which might be due to the higher acetate concentration. Acetate had a role as an antimicrobial substance that was reported to inhibit yeast effectively during aerobic exposure [28]. During cold conditions, the yeast and mold grow slowly. This indicates that during cold conditions (winter to spring), farmers do not have to worry about the aerobic stability of FTMR as the feed-out period is commonly 2 to 3 d. This inhibition of yeast growth was also supported with the result of pH at 7 d of aerobic exposure, where the MIX diets had a lower pH than the CON diets. The yeast inhibition in the present study confirmed that application of corn silage with dual-purpose LAB as a microbial source confirmed an antifungal activity on FTMR. The better fermentation characteristics in all diets with low temperature conditions after silo opening might be a reason why the inoculant and diet level had no effect on the growth of *Bacillus* during aerobic exposure.

In terms of rumen fermentation kinetics estimated from *in vitro* gas production, the application of corn silage with dual-purpose LAB confirmed carboxylesterase activity in FTMR through the improvement of the potentially fermentable fraction and the total fermentable fraction. These results agreed with previous studies that reported the digestibility improvement caused by carboxylesterase-producing LAB or direct ferulic-acid esterase in silage [11,12,14]. The presence of carboxylesterase during fermentation could degrade the lignin complex and might provide more soluble carbohydrate [7,8]. This process could increase the immediately fermentable fraction same as in the LOW diet. However, this beneficial effect did not occur in HIGH diet due to the low structural carbohydrate concentration. The rumen pH was lower by the MIX diets, which was affected by the higher total VFA concentration. The increase of total VFA concentration supported the results of IVDMD, IVNDFD, and the rumen fermentation kinetics in the present study that also indicated the presence of *L. brevis* 5M2 and *L. buchneri* 6M1 in the fermentation of FTMR. Generally, the HIGH diet provided a higher soluble carbohydrate with a lower structural carbohydrate that caused higher rumen fermentation kinetics and its fermentation indices than the LOW diet [30].

## CONCLUSION

The present study concluded that corn silage with dual-purpose LAB as a microbial source confirmed antifungal and carboxylesterase effects on FTMR. These results indicated that *L. brevis* 5M2 and *L. buchneri* 6M1 from corn silage controlled the fermentation of FTMR. The interaction effect between dietary energy level and inoculant only influenced the immediately fermentable fraction in the rumen, which the inoculant effect was effective with the low energy diet. The present study also concluded that feedstuff applied LAB as a ration ingredient could be a source of microbial inoculant to improve the quality of fermented diets.

## Figures and Tables

**Table 1:** Ingredients of low and high energy diets used in the present study (%, as fed)

Item	LOW^1)^		HIGH	
	
	CON	MIX	CON	MIX
Corn silage	14.5	14.5	11.1	11.1
Sudangrass silage	12.1	12.1	9.20	9.20
Rice straw	25.2	25.2	19.2	19.2
Corn meal	31.7	31.7	26.4	26.4
Corn gluten feed	8.49	8.49	11.7	11.7
Soybean meal	4.64	4.64	5.15	5.15
Lupin seed	2.41	2.41	5.87	5.87
Palm kernel	0.00	0.00	7.58	7.58
DDGS	0.00	0.00	1.90	1.90
Molasses	0.48	0.48	0.72	0.72
Premix^2)^	0.50	0.50	1.19	1.19

1) LOW, low energy diet; HIGH, high energy diet; CON, fermented total mixed ration with corn silage applied no inoculant; MIX, fermented total mixed ration with corn silage applied inoculant mixture of *L. brevis* 5M2 and *L. buchneri* 6M1.

2) One kilogram contained the following: vitamin A, 450,000 IU; vitamin D_3_, 45,000 IU; vitamin E, 1,500 IU; pantothenic acid, 40 mg; niacin, 30 mg; biotin, 20 mg; folic acid, 10 mg; FeSO_4_, 3,600 mg; CoSO_4_, 150 mg; CuSO_4_, 4,500 mg; MnSO_4_, 1,500 mg; ZnSO_4_, 2,200 mg; I, 400 mg; Se (Na), 150 mg; limestone, 2,000 mg; salt, 650 mg.

**Table 2:** Chemical compositions, *in vitro* nutrient digestibilities, and total digestible nutrients of low and high diets just before ensiling (%, DM)

Item	LOW^1)^		HIGH		SEM	Contrast^2)^		
		
	CON	MIX	CON	MIX		INO	DIET	INO×DIET
Dry matter	65.3^b^	65.3^b^	68.5^a^	68.4^a^	0.142	0.768	<0.001	0.860
Crude protein	12.7^b^	12.6^b^	13.4^a^	13.5^a^	0.160	0.857	<0.001	0.226
Ether extract	3.27^a^	3.35^a^	2.70^b^	2.86^b^	0.332	0.506	0.009	0.838
Crude ash	12.0^a^	12.0^a^	11.0^b^	10.5^b^	0.353	0.279	<0.001	0.279
Neutral detergent fiber	42.3^a^	42.4^a^	32.4^b^	32.5^b^	0.294	0.658	<0.001	0.706
Acid detergent fiber	22.3^a^	22.0^a^	14.9^b^	14.9^b^	0.381	0.540	<0.001	0.578
Hemicellulose	20.0^a^	20.3^a^	17.5^b^	17.6^b^	0.446	0.893	<0.001	0.933
IVDMD	70.5^b^	70.4^b^	74.0^a^	73.9^a^	0.962	0.907	<0.001	0.954
IVNDFD	50.3^b^	49.7^b^	52.7^a^	52.3^a^	0.543	0.242	0.001	0.931
TDN	71.3^b^	71.2^b^	77.1^a^	77.0^a^	0.114	0.946	<0.001	0.975

SEM, standard error of mean; DM, dry matter; IVDMD, *in vitro* dry matter digestibility; IVNDFD, *in vitro* neutral detergent fiber digestibility; TDN, total digestible nutrients.

1) LOW, low energy diet; HIGH, high energy diet; CON, fermented total mixed ration with corn silage applied no inoculant; MIX, fermented total mixed ration with corn silage applied inoculant mixture of *L. brevis* 5M2 and *L. buchneri* 6M1.

2) INO, inoculant effect; DIET, diet level effect; INO×DIET, interaction effect between inoculant and diet level.

a,b Means in the same row with different superscripts differ significantly (p<0.05).

**Table 3:** Effect of corn silage applied antifungal and carboxylesterase inoculant on chemical composition of total mixed ration fermented for 40 d (%, DM)

Item	LOW^1)^		HIGH		SEM	Contrast^2)^		
		
	CON	MIX	CON	MIX		INO	DIET	INO×DIET
Dry matter	64.0^b^	64.2^b^	67.5^a^	67.4^a^	0.488	0.946	<0.001	0.900
Crude protein	10.9^b^	11.0^b^	12.7^a^	13.0^a^	0.260	0.071	<0.001	0.444
Ether extract	3.40^a^	3.37^a^	2.78^b^	2.73^b^	0.144	0.646	<0.001	0.892
Crude ash	11.5^a^	11.6^a^	10.1^b^	9.91^b^	0.572	0.644	<0.001	0.841
Neutral detergent fiber	44.8^a^	45.0^a^	33.5^b^	33.0^b^	2.535	0.908	<0.001	0.787
Acid detergent fiber	23.7^a^	21.7^a^	16.0^b^	15.2^b^	0.928	0.032	<0.001	0.291
Hemicellulose	21.1^a^	23.3^a^	17.5^b^	17.7^b^	0.954	0.104	<0.001	0.216
IVDMD	59.9^d^	62.0^c^	65.9^b^	69.0^a^	0.501	<0.001	<0.001	0.122
IVNDFD	49.7^d^	52.2^c^	55.2^b^	58.5^a^	0.789	0.001	<0.001	0.415

DM, dry matter; SEM, standard error of mean; IVDMD, *in vitro* dry matter digestibility; IVNDFD, *in vitro* neutral detergent fiber digestibility.

1) LOW, low energy diet; HIGH, high energy diet; CON, fermented total mixed ration with corn silage applied no inoculant; MIX, fermented total mixed ration with corn silage applied inoculant mixture of *L. brevis* 5M2 and *L. buchneri* 6M1.

2) INO, inoculant effect; DIET, diet level effect; INO×DIET, interaction effect between inoculant and diet level.

a–d Means in the same row with different superscripts differ significantly (p<0.05).

**Table 4:** Effect of corn silage applied antifungal and carboxylesterase inoculant on fermentation characteristics and microbial counts of total mixed ration fermented for 40 d

Item	LOW^1)^		HIGH		SEM	Contrast^2)^		
		
	CON	MIX	CON	MIX		INO	DIET	INO×DIET
Fermentation indices								
pH	5.04^b^	5.03^b^	5.24^a^	5.26^a^	0.035	0.779	<0.001	0.383
Ammonia-N (% DM)	0.04^b^	0.04^b^	0.07^a^	0.06^a^	0.003	0.524	<0.001	1.000
Lactate (% DM)	1.57^ab^	1.49^b^	1.78^a^	1.66^ab^	0.100	0.157	0.013	0.773
Acetate (% DM)	2.13^b^	2.38^b^	3.79^a^	4.01^a^	0.072	0.010	<0.001	0.748
Propionate (% DM)	ND	ND	ND	ND	NA	NA	NA	NA
Butyrate (% DM)	ND	ND	ND	ND	NA	NA	NA	NA
Lactate:acetate	0.74^a^	0.63^a^	0.47^b^	0.41^b^	0.045	0.078	<0.001	0.142
Microbial counts (log10 cfu/g)								
Lactic acid bacteria	7.75^a^	7.88^a^	7.57^b^	7.65^b^	0.156	0.240	0.036	0.757
* Bacillus*	7.44	7.40	7.44	7.39	0.111	0.471	0.966	0.883
Yeast	7.63^a^	7.68^a^	7.43^b^	7.41^b^	0.123	0.871	0.005	0.587
Mold	ND	ND	ND	ND	NA	NA	NA	NA

SEM, standard error of mean; DM, dry matter; ND, not detected; cfu, colony forming unit; NA, not applicable.

1) LOW, low energy diet; HIGH, high energy diet; CON, fermented total mixed ration with corn silage applied no inoculant; MIX, fermented total mixed ration with corn silage applied inoculant mixture of *L. brevis* 5M2 and *L. buchneri* 6M1.

2) INO, inoculant effect; DIET, diet level effect; INO×DIET, interaction effect between inoculant and diet level.

a,b Means in the same row with different superscripts differ significantly (p<0.05).

**Table 5:** Effect of corn silage applied antifungal and carboxylesterase inoculant on mean of pH and microbial count from fermented total mixed ration during aerobic exposure

Item	LOW^1)^		HIGH		SEM	Contrast^2)^		
		
	CON	MIX	CON	MIX		INO	DIET	INO×DIET
pH	5.04^b^	5.02^b^	5.26^a^	5.27^a^	0.025	<0.001	0.556	0.066
Microbial counts (log10 cfu/g)								
Lactic acid bacteria	7.24^ab^	7.41^a^	7.03^b^	7.20^b^	0.106	0.001	0.013	0.979
* Bacillus*	7.51	7.53	7.51	7.49	0.069	0.598	0.972	0.647
Yeast	7.77^a^	7.76^a^	7.55^b^	7.54^b^	0.026	<0.001	0.342	0.451

SEM, standard error of mean; cfu, colony forming unit.

1) LOW, low energy diet; HIGH, high energy diet; CON, fermented total mixed ration with corn silage applied no inoculant; MIX, fermented total mixed ration with corn silage applied inoculant mixture of *L. brevis* 5M2 and *L. buchneri* 6M1.

2) INO, inoculant effect; DIET, diet level effect; INO×DIET, interaction effect between inoculant and diet level.

a,b Means in the same row with different superscripts differ significantly (p<0.05).

**Table 6:** Effect of corn silage applied antifungal and carboxylesterase inoculant on fermentation kinetics estimated from *in vitro* gas production of fermented total mixed ration incubated for 48 h with rumen buffer

Item^1)^	LOW^2)^		HIGH		SEM	Contrast^3)^		
		
	CON	MIX	CON	MIX		INO	DIET	INO×DIET
A (mL/g DM)	1.77^b^	1.95^ab^	2.00^a^	1.93^ab^	0.089	0.699	0.041	0.036
B (mL/g DM)	2.36^b^	2.47^b^	3.12^a^	3.49^a^	0.166	0.018	<0.001	0.221
A+B (mL/g DM)	4.16^b^	4.41^b^	5.12^a^	5.42^a^	0.143	0.009	<0.001	0.786
C (%/h)	0.22	0.21	0.21	0.19	0.017	0.141	0.204	0.558
L (h)	4.66^a^	4.74^a^	4.23^b^	3.76^b^	0.239	0.070	<0.001	0.095

DM, dry matter; SEM, standard error of mean.

1) A, the immediately fermentable fraction; B, the potentially fermentable fraction; A+B, the total fermentable fraction; C, the fractional fermentation rate; L, the lag phase.

2) LOW, low energy diet; HIGH, high energy diet; CON, fermented total mixed ration with corn silage applied no inoculant; MIX, fermented total mixed ration with corn silage applied inoculant mixture of *L. brevis* 5M2 and *L. buchneri* 6M1.

3) INO, inoculant effect; DIET, diet level effect; INO×DIET, interaction effect between inoculant and diet level.

a,b Means in the same row with different superscripts differ significantly (p<0.05).

**Table 7:** Effect of corn silage applied antifungal and carboxylesterase inoculant on rumen fermentation indices of fermented total mixed ration incubated for 48 h with rumen buffer

Item	LOW^1)^		HIGH		SEM	Contrast^2)^		
		
	CON	MIX	CON	MIX		INO	DIET	INO×DIET
pH	6.88^a^	6.84^ab^	6.75^bc^	6.68^c^	0.042	0.034	<0.001	0.397
Ammonia-N (mg/dL)	24.7^ab^	23.6^b^	26.4^a^	25.4^ab^	1.202	0.091	0.004	0.793
Total VFA (mM/L)	107.9^b^	114.4^b^	119.4^ab^	129.2^a^	5.042	0.014	0.001	0.584
Acetate, % molar	67.4	67.5	66.5	66.5	0.755	0.875	0.033	0.892
Propionate	16.9	16.7	16.8	17.0	0.858	0.990	0.839	0.713
Iso-butyrate	0.94	1.09	0.98	0.93	0.115	0.452	0.250	0.116
Butyrate	11.5	11.8	12.9	12.9	0.671	0.681	0.005	0.695
Iso-valerate	3.27^a^	2.88^ab^	2.81^ab^	2.67^b^	0.210	0.040	0.018	0.265
Acetate:propionate	3.99	4.03	3.95	3.93	0.299	0.950	0.551	0.772

SEM, standard error of mean; VFA, volatile fatty acid.

1) LOW, low energy diet; HIGH, high energy diet; CON, fermented total mixed ration with corn silage applied no inoculant; MIX, fermented total mixed ration with corn silage applied inoculant mixture of *L. brevis* 5M2 and *L. buchneri* 6M1.

2) INO, inoculant effect; DIET, diet level effect; INO×DIET, interaction effect between inoculant and diet level.

a–c Means in the same row with different superscripts differ significantly (p<0.05).

## SUPPLEMENTARY MATERIAL

**Supplementary Table S1**. Changes of pH (A), lactic acid bacteria (B), Bacillus (C), and yeast (D) of fermented total mixed ration during aerobic exposure
