# Supplementary material for: Antifungal and carboxylesterase-producing bacteria applied into corn silage still affected the fermented total mixed ration
Source: Anim Biosci. 2022 Nov 14;36(5):720–30. doi: 10.5713/ab.22.0232 (PMC10164483; doi:10.5713/ab.22.0232)
Supplement: Supplementary file 1 [file ab-22-0232-Supplementary-Table-1.pdf]

Supplementary Table S1. Changes of pH (A), lactic acid bacteria (B), *Bacillus* (C), and yeast (D) of fermented total mixed ration during aerobic exposure

Contrast[1)]	pH	LAB	*Bacillus*	Yeast
INO	0.045	0.001	0.898	0.478
DIET	<.001	<.001	0.396	<.001
DAY	0.007	<.001	0.004	<.001
INO*DIET	0.048	0.959	0.492	0.944
INO*DAY	0.500	0.023	0.893	0.396
DIET*DAY	0.076	0.684	0.866	0.800
INO*DIET*DAY	0.895	0.998	0.963	0.978
DAY linear	0.438	<.001	<.001	<.001
DAY quadratic	0.962	0.065	0.193	0.720

[1)] INO, inoculant effect; DIET, diet level effect; DAY, aerobic day effect; INO*DIET, interaction effect between inoculant and diet level; INO*HOUR, interaction effect between inoculant and aerobic day; DIET*HOUR, interaction effect between diet level and aerobic day; INO*DIET*HOUR, interaction effect between inoculant, diet level and aerobic day; DAY linear, linear effect of aerobic day; and DAY quadratic, quadratic effect of aerobic day.
